# Efficacy of risk stratification protocols and clinical, physical, and biochemical parameters to previse signals and symptoms during cardiovascular rehabilitation programs: Protocol for an observational trial

**DOI:** 10.1097/MD.0000000000015700

**Published:** 2019-06-14

**Authors:** Laís Manata Vanzella, Carolina Takahashi, Felipe Ribeiro, Isabelle Maina Lima, Anne Kastelianne França da Silva, Diego Giulliano Destro Christófaro, Luiz Carlos Marques Vanderlei

**Affiliations:** São Paulo State University (UNESP), School of Sciences and Technology, Presidente Prudente, São Paulo, Brazil.

**Keywords:** autonomic modulation, blood pressure, cytokines, exercise, heart rate, inflammation, muscle strength, rehabilitation services, risk, signals and symptoms

## Abstract

**Introduction:**

Despite the benefits, cardiovascular rehabilitation programs (CRPs) have been related to the appearance of signals and symptoms. Risk stratification protocols are commonly used to identify risks during the physical exercise; however, studies that investigate their efficacy to previse signals and symptoms are inconclusive. Furthermore, clinical, physical, and biochemical parameters have been used as risk markers for the appearance of adverse events, and to investigate their efficacy to previse signals and symptoms during the CRP sessions that could better guide the strategies adopted on these programs.

**Objectives:**

The aim of this study was to evaluate the correlations between risk stratification protocols and clinical, physical, and biochemical parameters with the appearance of signals/symptoms during CRP, as well as to evaluate if modifications on clinical, physical, and biochemical parameters could influence in the appearance of signals/symptoms during CRP.

**Materials and methods:**

The study was prospectively registered at ClinicalTrials.gov (NCT03446742). Forty-four patient participants of a CRP will be evaluated. First, their risk stratification is going to be performed by 2 evaluators and their clinical, physical, and biochemical parameters are going to be measured. Then, the patients are going to be followed during 24 sessions during their CRP routines in order to identify appearance of their signals/symptoms. So, the patients are going to perform their cardiovascular rehabilitation routines for 6 months and then, their clinical, physical, and biochemical parameters are going to be measured again and they are going to be followed during 24 sessions during their CRP routines in order to identify the appearance of their signals/symptoms.

## 1 Introduction

Cardiovascular diseases (CVDs) are considered the main cause of death in the world and their appearance is associated with changes^[1–3]^ that directly compromise the quality of life.^[4]^ Therefore, strategies to treat and prevent CVD are fundamental.

Cardiovascular rehabilitation programs (CRPs) are highlighted as an efficient way to prevent and treat CVD, mainly because of its beneficial effects.^[5–8]^ However, during the physical activity practice, a metabolic demand increase happens and promotes changes in the organism,^[9,10]^ which can facilitate the occurrence of signals and symptoms commonly found in CRP.^[11]^ In this context, to investigate factors that could previse the appearance of signals and symptoms during the CRP, would better guide the strategies adopted for its performance.

The literature has shown that clinical, physical, and biochemical parameters, such as heart rate variability (HRV),^[4,12–15]^ cardiorespiratory parameters,^[16–19]^ functional capacity (FC), muscle strength (MS),^[20–23]^ and inflammatory cytokines,^[24,25]^ have been used as a risk marker of mortality, cardiovascular complications, and adverse events on different population, and that some of these parameters are also correlated with specific responses during the physical exercise.^[26–31]^ However, a literature research did not point studies that analyzed whether these parameters could be used as a risk predictor of signals and symptoms during the CRP session. Furthermore, these parameters can directly influence the intensity of these alterations promoted by the exercise and, consequently, be related with the appearance of signals and symptoms.

It is important to highlight that patients submitted to a CRP generally have their risk stratified through different protocols, which allows the practitioner to identify their level of cardiovascular risk.^[32]^ However, some of these protocols efficacy to previse serious complications during the CRP did not obtain significative results.^[33–35]^

Taken together, these data point to some gaps in the literature. How are the risk stratification protocols effective to previse signals and symptoms during the CRP? Can clinical, physical, and biochemical parameters be used to previse signals and symptoms during the CRP? If yes, is it possible to establish a cut-off point for these parameters that can better previse the appearance of signals and symptoms in these programs? Are changes in clinical, physical, or biochemical parameters induced by CRP accompanied by changes in the appearance of signals and symptoms during the CRP?

This study was designed to answer these questions: to evaluate the risk stratification protocols efficacy to previse signals and symptoms during the CRP; to analyze the correlation between clinical, physical, and biochemical parameters, measured at rest, with the presence of signals/symptoms in CRP participants; and to evaluate whether changes in clinical, physical, or biochemical parameters, induced by CRP, will influence the appearance of signals and symptoms during these programs.

## 2 Materials and methods

This is a longitudinal observational study of a cohort, registered prospectively on ClinicalTrials.gov (NCT03446742). It going to be developed with a convenience sample of patients regularly attending a CRP based on exercise.

### 2.1 Eligibility criteria

Subjects of both genders, with clinical diagnosis of CVD and/or cardiovascular risk factor, able to realize all the evaluations proposed and in accordance to the study procedures are going to be included. Patients with less than 30% of attendance during the period of the protocol are going to be excluded.

### 2.2 Sample size

The sample size was based on rMSSD index^[36]^ because it presents the highest sample number necessary for this study, compared with the other main variables [6 minutes walking test^[37]^ and tumor necrosis factor (TNF)-alpha^[38]^]. A standard deviation of 17 ms, alpha risk of 5%, and beta of 80% was considered for the calculus, which resulted in 44 subjects.

### 2.3 Ethical aspects

The volunteers are going to be previously informed about the objectives and procedures of the study and, after agreeing to participate, they will sign a consent form. All the procedures that are going to be used in the study were approved by the Committee for Ethics and Research of the São Paulo State University (UNESP), School of Technology and Sciences, Presidente Prudente (CAAE: 79213417.0.0000.5402).

### 2.4 Study design

This study is going to be divided in 3 phases. Initially, all the volunteers are going to have their medical records analyzed, and information about the sample characterization and medical examinations are going to be extracted. Two independent blinded and trained measurers are going to realize the risk stratification of the volunteers, using the protocols described into the literature review of Silva et al.^[39]^ Following this, clinical (cardiorespiratory parameters and autonomic modulation), physical [maximum isometric resistance (MIR), maximum isometric voluntary contraction (MIVC), cardiorespiratory fitness, and physical activity level], and biochemical [interleukin (IL)-6, TNF-alpha, and IL-10] parameters are going to be evaluated by a blinded and trained measurer and the subjects are going to be accompanied during 24 sessions of their CRP routines, to register the appearance of their signals and symptoms.

In the second phase, the volunteers are going to realize their normal CRP routines for 6 months. In the third phase, the clinical, physical, and biochemical parameters, described above, are going to be evaluated by a blinded and trained measurer and the volunteers are going to be accompanied for more 24 sessions of their CRP routines, to register the appearance of their signals and symptoms as well.

**Figure 1:**
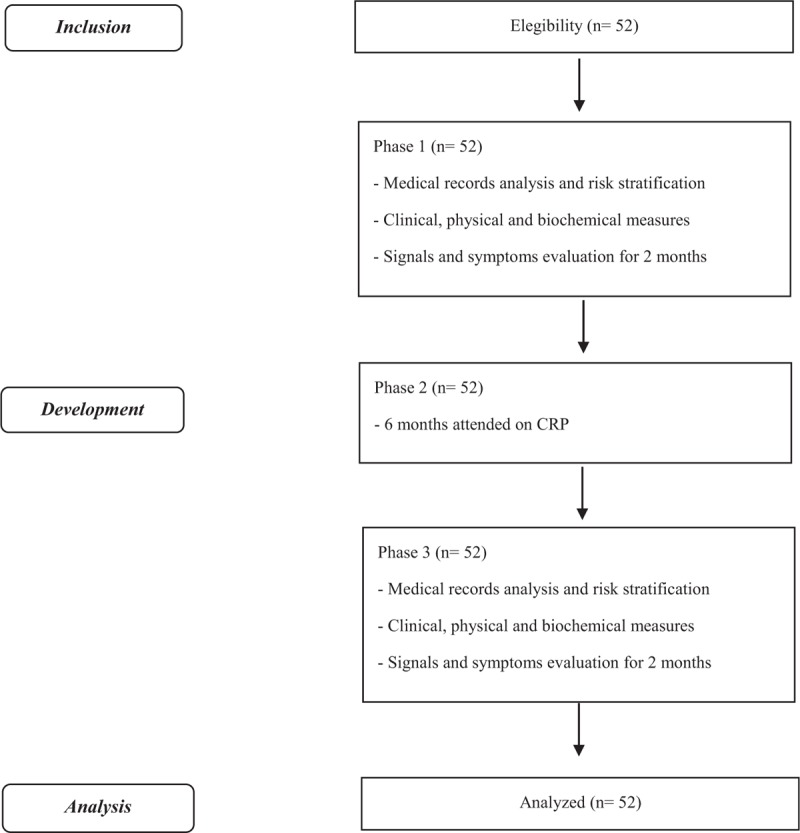
Flowchart. CRP = cardiovascular rehabilitation program.

The description of the phases and the volunteers that will be included in each one of them are reported at Fig. 1.

### 2.5 Sample characterization

For the sample characterization, the volunteers’ medical records are going to be analyzed in order to identify information about their age, gender, clinical diagnoses, cardiovascular risk factors, and associated pathologies (musculoskeletal, neurological, pulmonary, and/or metabolic disfunction).

For the volunteers’ risk stratification according to the protocols selected in this study, their clinical history and the results of their last laboratory and complementary examinations related to the cardiovascular system are going to be extracted from their medical records.

For the clinical history, information about the number of cardiorespiratory arrests, hospital days, and complications during the hospital days or after procedures are going to be considered. For the laboratory tests, blood glucose, triglycerides, total cholesterol, high-density lipoprotein (HDL)-cholesterol, and low-density lipoprotein (LDL)-cholesterol are going to be considered. For the complementary examinations, cardiovascular examinations, such as exercise stress test, echocardiogram, Holter, catheterization, myocardial scintigraph, electrocardiogram, and coronary angiotomography are going to be considered.

We are also going to measure the height in orthostatic position with a stadiometer (Sanny, Personal Caprice, Brazil) and body mass with a digital balance (Balmak, Premium, Bk–200Fa, Brazil), in order to calculate the body mass index (BMI).^[40]^

### 2.6 Cardiac risk stratification

For the volunteers’ cardiac risk stratification, 8 protocols reported by Silva et al^[39]^ are going to be used. The patients are going to be classified in accordance to each protocol, considering their tests and evaluation, as less, moderate, and high risk, and if they have 1 characteristic considered as higher cardiovascular risk, they will be classified on this category. Furthermore, if the patients do not fulfill the criteria for the stratification in any of the protocols, they are going to be classified as “impossible to classify.”

The American Heart Association (AHA)^[41]^ protocol classifies the patients according to risk classes (A, B, C, D), considering the presence of symptoms or CVD, respiratory rate, and the exercise stress test. The patients classified as A risk class are going to be considered as less risk, B risk class are going to be considered as moderate risk, and C risk class are going to be considered as high risk. According to AHA, patients classified as C risk class should not participate in CRP.

### 2.7 Evaluation of clinical parameters

In relation to clinical parameters, we are going to evaluate some of them related to the cardiorespiratory system and autonomic modulation, such as blood pressure (BP), heart rate (HR), oxygen saturation (SatO_2_), respiratory rate (f), maximal inspiratory pressure (MIP), maximal expiratory pressure (MEP), peak expiratory flow (PEF), forced expiratory volume in 1 second (FEV1), forced vital capacity (FVC), FEV1/FVC ratio, and HRV.

#### 2.7.1 Cardiovascular parameters

The blood pressure measure is going to be done as an indirect form, with an stethoscope (Littmann, St Paul, MN) and aneroid sphygmomanometer (Welch Allyn, Tycos, NY).^[42]^ The HR is going to be verified using a cardiac monitor Polar (Polar Electro OY, V800, Finland) and its value is going to be obtained through the average of the 5 to 20 minutes of the RR intervals.

#### 2.7.2 Respiratory parameters

For the oxygen saturation, we are going to use a pulse oximeter (Rossmax Innotek Corp, SB220, Taiwan), which is going to be fixed on the index finger of each volunteer. The respiratory rate is going to be measured individually through the number of the respiratory cycles performed for one minute.

The MIP and MEP are going to be measured with an analogical manovacuometer (Murenas, 71001WTB CL1.6, Brasil), calibrated in 300 cmH_2_O.^[43,44]^ To measure the spirometry values, we are going to use a portable spirometry (MIR Medical International Research, Spirobank II Advanced, Itália). Through the time-volume curve, the peak expiratory flor (PEF), FEV1, FVC, and FEV1/FVC^[45]^ are going to be obtained.

#### 2.7.3 Autonomic modulation

The autonomic modulation is going to be evaluated through the HRV analysis. For this analysis, the HR will be recorded beat-to-beat in a quiet room, with temperature controlled between 21° and 24° C and air relative humidity between 40% and 60%. The data collection is going to be done individually and the patients remain at rest in an orthostatic position for 30 minutes. The patients are going to be oriented to not consuming stimulant substances of the ANS such as alcoholic drinks, coffee, tea, soda, and chocolate for 24 hours before HRV analysis.

A heart rate monitor Polar RS800CX (Polar Electro OY, V800, Finland) is going to be used for HR record, and the data recorded are going to be transferred to the computer through the Polar Flow web service (https://flow.polar.com/).

For the data analysis, we are going to use 1000 consecutive RR intervals, after a digital filtering complemented by a manual filtering, to eliminate ectopic, premature, and artifacts beats, and only series with more than 95% of heart sinus beat are going to be included in the study.

Linear indices of HRV in the time and frequency domain are going to be used for HRV analysis. In the time domain, RMSSD (root mean square to the successive differences between each heartbeat) and SDNN (standard deviation of all normal RR intervals) are going to be calculated.^[46]^ In the frequency domain, the spectral components of low frequency (LF: 0.04–0.15 Hz) and high frequency (HF: 0.15–0.40 Hz) are going to be evaluated, in milliseconds squared (ms^2^) and normalized units (nu), and also the relation between LF and HF components (LF/HF). The spectral analyses are going to be calculated using the algorithm of Fast Fourier transform.^[46]^

In addition, the HRV is going to be also calculated through the quantitative analysis of Poincaré Plot, considering the following indices: SD1 (standard deviation of distances of diagonal points), SD2 (standard deviation of the distances from points to lines), and finally the SD1/SD2 that describes the ratio between short and long variations of RR intervals.^[46]^

All the HRV indices are going to be obtained using Kubios HRV Standard software – version 3.0.0 (Kubios, Biosignal Analysis and Medical Image Group, Department of Physics, University of Kuopio, Kuopio, Finland).

### 2.8 Physical parameters

For analysis of physical parameters, the MIR, MIVC, cardiorespiratory fitness, and physical activity level are going to be measured.

#### 2.8.1 Maximum isometric voluntary contraction (MIVC)

The maximal MS is going to be evaluated using a MIVC, performed at the isokinetic dynamometer (Biodex system 4 Pro; New York).^[47]^ Before the beginning of the test, the equipment is going to be calibrated and the patients positioned with individual measures, which are going to be maintained for all moments. The equipment has bands that are going to be fixed on the patients in the trunk, hip, thigh, and distal area of the lower limb (dominant) in order to isolate knee movement. The dominant limb is going to be determined using the following question: “Which leg do you use to kick a ball?”

Before the test, the patients are going to undergo a warm up, consisting of 10 repetitions of concentric contraction of knee flexion-extension at 180°/s throughout the range of motion. The MS is going to be measured throughout the higher torque value obtained among three 5-second repetitions of MIVC at 60° of knee flexion (with 0° corresponding to the maximum extension). To minimize possible fatigue effects, the volunteers are going to have 2 minutes of rest between the test repetitions. Furthermore, the patients are going to be instructed to perform their maximum strength performance.

#### 2.8.2 Maximum isometric resistance (MIR)

Maximal isometric muscle resistance is going to be measured using the MIR, performed at the isokinetic dynamometer (Biodex Medical Systems, System 4 Pro, New York) with the same initial orientations of the CIVM.^[48]^

Initially, the patients are going to perform an isotonic warm-up, consisting of 10 knee flexion-extension at a speed of 330°/s, respecting a 90° amplitude. Next, an isometric warm-up is going to be performed, where the patients are going to do 30 seconds of isometric contraction, with an intensity determined according to Borg subjective perception of effort scale (5--6 of intensity).

After 2 minutes of rest, the patients are going to be familiarized with the isometric resistance test. For this, they are going to perform his/her highest perceived effort (according to Borg scale) and to maintain this effort for as long as possible until he/she is unable to maintain muscle contraction. Twenty-five hours after the familiarization, the patients are going to perform the MIR test, which consists of the same familiarization procedures described above.

In order to stimulate the patient, they are going to accompany, in the monitor coupled to the equipment, the force generation line that serves as a visual feedback. In addition, verbal stimuli are going to be given by the measurer during the test.

#### 2.8.3 Cardiorespiratory fitness

The cardiorespiratory fitness is going to be evaluated using a 6-minute walking test (6MWT).^[49]^ The patients are going to be encouraged by the measurer during the test to maintain the same walk rhythm until the end of its. The test is going to be performed twice, with 30 minutes of interval between than, and the highest distance performed by the patient is going to be considered for the analysis.

#### 2.8.4 Physical activity level

The physical activity level is going to be measured day-by-day using a triaxial accelerometer Actigraph GT3X-BT (ActiGraph, LLC, Pensacola, FL),^[50,51]^ calibrated at 30 Hz. For this, the equipment is going to be positioned on the hip (dominant side) and the patients are going to use it for 7 days, withdrawing only during sleep and water activities.

To be considered a valid day, the individual should remain a period of 10 hours (600 minutes) with the equipment.^[15,50,51]^ After the use, counts activity are going to be analyzed using ActiLife 6.11.8 software (ActiGraph, LLC, Pensacola, FL) with 1 second of epoch, and later, in order to obtain the total in minutes of the time spent in moderate/vigorous physical activity data are going to be re-entered for 60-second epoch. Valid periods are going to be determined subtracting the 24 hours nonvalid periods.^[50]^ These data will provide information related to physical activity level (light, moderate, and high), total steps per day, and total counts per minute, which is estimated based on the following cutoff points: light intensity: 100 to 2019 counts; moderate 2020 to 5998 counts; and vigorous 5999 counts or more.

### 2.9 Biochemical parameters

The biochemical parameters IL-6, TNF-alpha, and IL-10 are going to be analyzed. For these, the blood sample (15 mL) is going to be collected with the subjects in fasting for 12 hours. The blood samples are going to be allocated into 2 vacutainer tubes (Becton Dickinson, BD, Juiz de Fora, MG Brazil) containing EDTA for plasma separation, centrifuged at 3500 g during 15 minutes at 4°C, and finally stored at -20°C for further analysis. Cytosines IL-6, IL-10, and TNF-alpha are going to be evaluated using the commercial ELISA kit (R&D Systems, Minneapolis, MN).

### 2.10 Cardiac rehabilitation program

The CRP applied to patients who are going to be recruited for the study is composed of the following steps: initial rest, warm-up, resistance, and relaxation phase. The program is performed 3 times a week (Mondays, Wednesdays, and Fridays) totalizing 60 minutes per day, divided as follows: 5 minutes of initial rest, which aims at assessing the BP, HR, and signals and symptoms commonly found in subjects with CVD; 15 minutes of warm-up, performing overall stretching, lower limb exercises, upper limb exercises, and exercises combining both modes; 30 minutes of resistance phase, performing individualized aerobic exercise according to their HR reserve values,^[52]^ using bicycle and treadmill (15 minutes of exercise in each one of these ergometers). In both ergometers, the HR is measured at the 4th and 10th minute, and in bicycle, the BP is also measured. If the patient has some physical restriction, the protocol will be performed in only one of the ergometers (in this case, the HR and BP are measured at the 5th, 15th, and 25th minute); 10 minutes of cooling down, performing a cardiovascular deceleration, for example, with few laps around the room, and finally stay at rest. At the end of this phase, the HR is measured and, if necessary, so is the BP.

### 2.11 Signals and symptoms identification

For the identification of the signals and symptoms during the CRP, a record is going to be prepared for each patient, containing the additional personal data, clinical diagnosis, use of medicines, and the signals and symptoms commonly found during CRP.

The most prevalent signals and symptoms found during CRP are symptoms: fatigue, muscle pain, angina, dizziness, nausea, and cramp; and signals: changes in pulse rate, increased systolic blood pressure during the exercise, increased diastolic blood pressure during the exercise, tachypnea, and pallor.

The signals are going to be observed and identified by previously trained professionals, and the symptoms are going to be referred to and/or confirmed by the patient at the end of the session. The observation of signals and symptoms is going to be performed during all phases of the session (initial rest, warm-up, resistance, and cooling down).

When the changes in pulse rate have been observed, the patients are going to perform the session with an electrocardiogram and the tracing is going to be recorded and analyzed to identify the rhythm disorder presented.

The evaluation of signals and symptoms is going to be performed during the 24 sessions. The symptomatology is going to be counted per session, regardless the number of times that the same signal/symptom occurs during the session.

### 2.12 Data analysis

The normality of data is going to be analyzed using Shapiro--Wilk test. According to the distribution, the population characteristics will be presented, as mean and standard deviation for parametric distribution and median and interquartile interval for nonparametric distribution.

The relationship between the number of signals and symptoms of the patients and the scale degree of the protocols (1, 2, and 3) is going to be analyzed using Pearson correlation. The concordance degree of each protocol with the number of signals and symptoms are going to be determined using coefficient of intraclass correlation. To determine the efficacy of the risk stratification protocols as soon as clinical, physical, and biochemical parameters to previse signals and symptoms during the CRP, analysis of sensibility and specificity using ROC curve is going to be done. The sensibility, specificity, and predictive value (positive and negative) for the occurrence of events are going to be also registered. If the values of the area on the curve are ≥0.650, it will be considered significative.

To evaluate whether the clinical, physical, and biochemical modifications have influenced in the occurrence of signals and symptoms, the sphericity of the data will be verified by Mauchly test, and if the sphericity be violated, Greenhouse--Geiser correction will be performed. The comparison between the different moments of the clinical (cardiorespiratory parameters and autonomic modulation), physical (maximum isometric endurance, MIVC, cardiorespiratory fitness and physical activity level), and biochemical (IL-6, TNF-alpha, and IL-10) are going to be realized by repeated measures analysis of variance (ANOVA) and the possible differences between the moments will be performed by Bonferroni post-hoc.

The statistical significance difference adopted is going to be fixed in 5% with 95% of confidence interval. The analysis is going to be done on SPSS 15.0 version (SPSS Inc., Chicago, IL).

## 3 Final considerations

As final considerations, we need to highlight the innovation of this study, which is going to be the first to investigate the efficacy of different stratification protocols and clinical, physical, and biochemical parameters to previse signals and symptoms during the CRP. Their realization will permit new study perspectives and to answer important questions that will help researchers and clinics that work with CRP. Considering the high prevalence of signals and symptoms observed in CRP, identifying the stratification protocols able to previse the appearance of these signals and symptoms will allow the physical therapist to define the most adequate risk stratification protocols, permitting more efficacy and security during these programs.

In addition, some patients who perform CRP do not have recent medical tests, which cannot enable an adequate risk stratification based on the stratification protocols proposed at the literature. In this sense, if clinical, physical, and biochemical variables, obtained at rest, will be related with the appearance of signals and symptoms during the CRP, it will also allow more efficacy and safety for the realization of CRP, mainly because these variables are at easy access and utilization.

## Acknowledgment

The authors would like to acknowledge the São Paulo research foundation - FAPESP, for supporting this research (2017/20657-5).

## Author contributions

**Conceptualization:** Laís Vanzella, Felipe Ribeiro, Anne Kastelianne França da Silva, Diego Giuliano Destro Christófaro, Luiz Carlos Marques Vanderlei.

**Data curation:** Laís Vanzella.

**Formal analysis:** Laís Vanzella, Diego Giuliano Destro Christófaro.

**Investigation:** Laís Vanzella, Anne Kastelianne França da Silva.

**Methodology:** Laís Vanzella, Carolina Takahashi, Felipe Ribeiro, Isabelle Maina Lima, Anne Kastelianne França da Silva, Diego Giuliano Destro Christófaro, Luiz Carlos Marques Vanderlei.

**Project administration:** Laís Vanzella, Felipe Ribeiro, Isabelle Maina Lima.

**Supervision:** Laís Vanzella, Carolina Takahashi, Luiz Carlos Marques Vanderlei.

**Writing – original draft:** Laís Vanzella, Luiz Carlos Marques Vanderlei.

**Writing – review & editing:** Laís Vanzella, Carolina Takahashi, Felipe Ribeiro, Isabelle Maina Lima, Anne Kastelianne França da Silva, Diego Giuliano Destro Christófaro, Luiz Carlos Marques Vanderlei.

Laís Vanzella orcid: 0000-0002-9494-3143.
